# A rare case report: multiple intrahepatic masses in a pediatric patient with citrin deficiency

**DOI:** 10.1007/s12672-024-01059-0

**Published:** 2024-05-31

**Authors:** Hui Lin, Hong Jiang, Qiang Chen, Xiang Pan, Mei Deng, Xiang-Ran Cai, Yuan-Zhi Lu, Yuan-Zong Song, Jun-Cheng Liu

**Affiliations:** 1 Department of Pediatrics, The First Affiliated Hospital, Jinan University, Guangzhou, 510632 China; 2 Department of Pediatric Surgery, The First Affiliated Hospital, Sun Yat-Sen University, Guangzhou, 510630 China; 3 Department of Pathology, The First Affiliated Hospital, Jinan University, Guangzhou, 510632 China; 4 Department of Radiology, The First Affiliated Hospital, Jinan University, Guangzhou, 510632 China

**Keywords:** Cholestasis, Citrin deficiency, Hepatoblastoma, *SLC25A13*, Variant

## Abstract

Deficiency of citrin, the liver-type aspartate-glutamate carrier, arises from biallelic mutations of the gene SLC25A13. Although citrin deficiency (CD) is associated with higher risk of hepatocellular carcinoma (HCC) in adult patients, this association remains inconclusive in pediatric cases. The patient in this paper had been diagnosed to have CD by SLC25A13 analysis at the age 10 months, and then in response to dietary therapy, her prolonged jaundice and marked hepatosplenomegaly resolved gradually. However, she was referred to the hospital once again due to recurrent abdominal distention for 2 weeks at her age 4 years and 9 months, when prominently enlarged liver and spleen were palpated, along with a strikingly elevated serum alpha-fetoprotein (AFP) level of 27605 ng/mL as well as a large mass in the right liver lobe and a suspected tumor thrombus within the portal vein on enhanced computed tomography. After 4 rounds of adjuvant chemotherapy, right hepatic lobectomy and portal venous embolectomy were performed at her age 5 years and 3 months, and metastatic hepatoblastoma was confirmed by histopathological analysis. Afterwards, the patient underwent 5 additional cycles of chemotherapy and her condition remained stable for 7 months after surgery. Unfortunately, hepatoblastoma recurred in the left lobe at the age 5 years and 10 months, which progressed rapidly into liver failure, and led to death at the age 6 years and 1 month. As far as we know, this is the the first case of hepatoblastoma in a patient with CD, raising the possibility of an association between these two conditions.

## Introduction

Citrin is a solute transporter protein mainly expressed in the mitochondrial inner membrane of the hepatocyte as the liver-type aspartate-glutamate carrier isoform 2, playing important roles in the malate-aspartate shuttle and urea cycle [1]. Citrin deficiency (CD) is an autosomal recessive disorder due to biallelic mutations of *SLC25A13* gene which was cloned in the year 1999 [2]. For this disorder, 3 age-dependent phenotypes have been reported thus far, i.e. Neonatal Intrahepatic Cholestasis caused by Citrin Deficiency (NICCD) in neonates or infants [3], adult-onset citrullinemia type II (CTLN2) in adolescents or adults, and Failure to Thrive and Dyslipidemia caused by Citrin Deficiency (FTTDCD) in older children [4, 5].

As an inborn error of metabolism, CD might have later complications. Actually, CTLN2 has been reported to be related to hepatocellular carcinoma (HCC) [6, 7], and the incidence of this malignancy was estimated to be approximately 8% in CTLN2 patients [8]. Thus far, however, this association remains inconclusive in pediatric patients due to lacking pathological evidences. This paper, by way of clinical, molecular and pathological analysis, described the first pediatric patient who suffered from CD complicated by hepatoblastoma with unfavorable outcome.

## Case presentation

A 9.3-month-old female was admitted to the Department of Pediatrics, the First Affiliated Hospital, Jinan University with the complaint of jaundiced skin over 9 months. The patient presented with sustained jaundice since 7 days after birth, but it was until her age 8.5 months that she was firstly referred to a local hospital where biochemistry test revealed raised serum levels of alanine transaminase (ALT), aspartate transaminase (AST), total bilirubin (TBIL), direct bilirubin (DBIL), and indirect bilirubin (IBIL). Then she was admitted to our hospital at the age 9.3 months, when physical examination revealed jaundiced skin and sclera, enlarged liver 6.5 cm below the right costal margin, and enlarged spleen 6 cm below the left costal margin. Moreover, markedly raised serum alpha-fetoprotein (AFP) was detected besides elevated ALT, AST, TBIL, DBIL, and IBIL (Table 1), indicating a cholestatic liver disease. On *SLC25A13* analysis at her age 10 months, the patient proved to be homozygous for the prevalent pathogenic mutation c.852_855del4, and thus NICCD was definitely diagnosed. Thereafter, a lactose-free and medium-chain triglycerides (MCT)-enriched formula was administered. She was discharged a month later, and her jaundice and hepatosplenomegaly resolved gradually on subsequent follow up in our clinic, and the liver function indices returned to normal at the age 1 years and 9 months (Table 1).

**Table 1:** Biochemical alterations over time in the patient with citrin deficiency

Indices (reference range)	Ages																		
	8.5 M	9.3 M	9.6 M	9.8 M	10.7 M	11.7 M	1Y3M	1Y9M	2Y8M	4Y	4Y 9 M	4Y 10 M	5Y 3 M^▲^	5Y 3 M^▼^	5Y 4 M	5Y 5 M	5Y 6 M	5Y 10 M	6Y
ALT (5–40U/L)	75	44	75	104	89	89	54	32	22	22	50	53	12	497	19	22	32	54	116
AST (5–40U/L)	189	191	206	212	148	140	57	44	38	31	72	105	32	266	44	46	44	57	258
GGT (8–50U/L)	279	91	163	154	245	289	101	69	28	28	60	110	64	43	25	26	29	152	279
ALP (40–500U/L)	402	834	765	810	718	642	435	358	217	247	219	211	140	107	181	224	217	132	387
TP (60.0–83.0 g/L)	62.3	67.7	64.5	68.7	68.1	69.7	64.7	69.8	67.3	69.7	72.8	75.2	84.1	65	67.7	78.6	76.6	59	–
Alb (35.0–55.0 g/L)	32.3	33.9	33.9	37.8	40.3	43.2	44.1	49.5	45.9	45.5	46.2	42.2	43.4	41	39	40.8	42.2	32	33
Glb (20.0–30.0 g/L)	30	33.8	30.6	30.9	27.8	26.5	20.6	20.3	21.4	24.2	26.6	33.0	40.7	24	44.5	37.8	34.4	27	–
Tbil (2–19 µmol/L)	46.3	90.1	42.0	37.2	16.8	12.9	5.3	5.2	5.0	5.9	7.5	4.6	17.6	33.7	27.2	30.1	12.8	15.6	633.3
Dbil (0–6 µmol/L)	34.6	49.4	28.3	22.8	10.3	6.6	2.3	1.8	0.9	1.3	1.7	1.6	1.7	0.0	3.4	2.8	3.4	0.0	497.6
Ibil (2.56–20.9 µmol/L)	11.7	40.7	13.7	14.4	6.5	6.3	3.0	3.4	4.1	4.6	5.8	3.0	15.9	22.1	23.8	27.3	9.4	8.9	135.7
TBA (0–10 µmol/L)	396	186	251.6	39.9	56.4	30.8	17.6	4.7	9.1	5.7	19.5	7.7	7.6	–	45.7	28.3	11.9	–	–
AFP (0–12 ng/ml)	–	26,778	22,153	14,797	2415	804	81	14	38	–	27605	134392	25530	5219	643	480	939	63866	–

▲Three days before surgery; ▼Three days after surgery. The ages “Y” and “M” represented years and months, respectively

*ALT* alanine aminotransferase, *AST* aspartate aminotransferase, *GGT* γ-glutamyl transpeptidase, *ALP* alkaline phosphatase, *TP* total protein, *Alb* albumin, *Glb* globulin, *Tbil* total bilirubin, *Dbil* direct bilirubin, *Ibil* indirect bilirubin, *TBA* total bile acids, *AFP* alpha-fetoprotein,—not tested

**Fig. 1:**
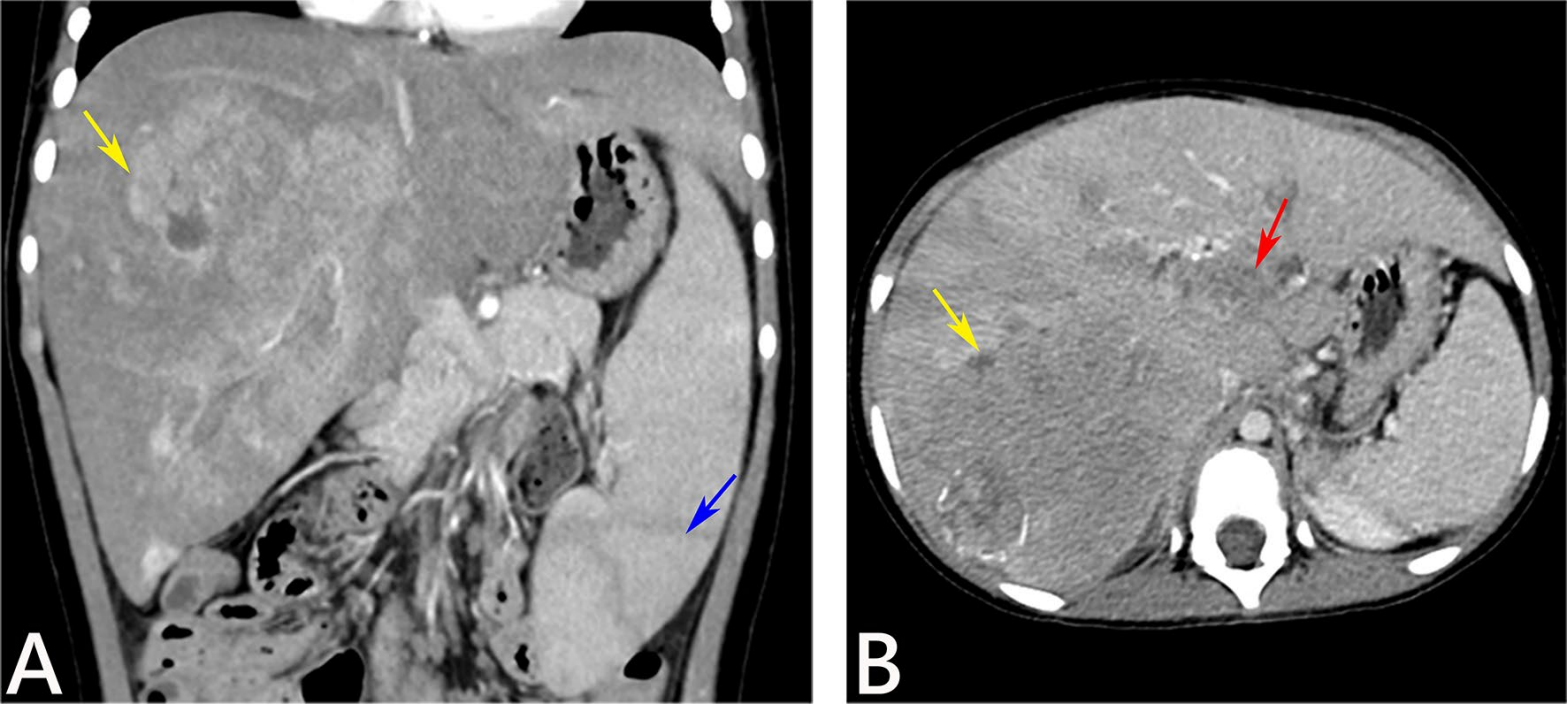
Abdominal enhanced CT findings of the patient at the age 4 years and 9 months. Coronal and axial contrast-enhanced CT images in arterial phase (**A**) demonstrated a large mass in the right lobe of the liver with the heterogeneous arterial enhancement (yellow arrow) and the enlarged spleen (blue arrow), while portal-venous phase (**B**) showed a large mass in the right lobe of the liver with wash out (yellow arrow) and a tumor thrombus within the portal vein (red arrow)

However, when aged 4 years and 9 months, she was referred to the hospital once again with the complaint of abdominal distention for 2 weeks. Physical examination at referral revealed a body temperature of 36.6 ℃, heart rate 102 beats/min (bpm), respiratory rate 21 bpm, weight 16.2 kg, height 102.7 cm, and head circumference 48.1 cm. No jaundice was observed in the skin and sclera. Respiratory and cardiac examination was unremarkable. A liver 7.0 cm below the right and a spleen 4.0 cm below the left costal margin, were palpable both in the mid clavicular line. Neurological exam was normal. The extremities were warm, and the distal perfusion was excellent. Laboratory tests showed a markedly elevated serum AFP level of 27,605 ng/ml (reference range:0–12 ng/mL), and ALT, AST, γ-glutamyl transpeptidase (GGT) and total bile acids (TBA) levels were also raised (Table 1). Abdominal ultrasonography revealed multiple strong echoes in the liver, with the largest one being 28 × 30 mm in size. Magnetic resonance imaging (MRI) showed a large mass of 82 × 95 × 78 mm in size in the right hepatic lobe and an embolus in the portal vein. Further enhanced computed tomography (CT) revealed multiple masses in the right hepatic lobe with the largest one 97 × 85 × 89 mm in size; a suspected tumor embolus within the portal vein and an enlarged spleen were also observed (Fig. 1).

**Fig. 2:**
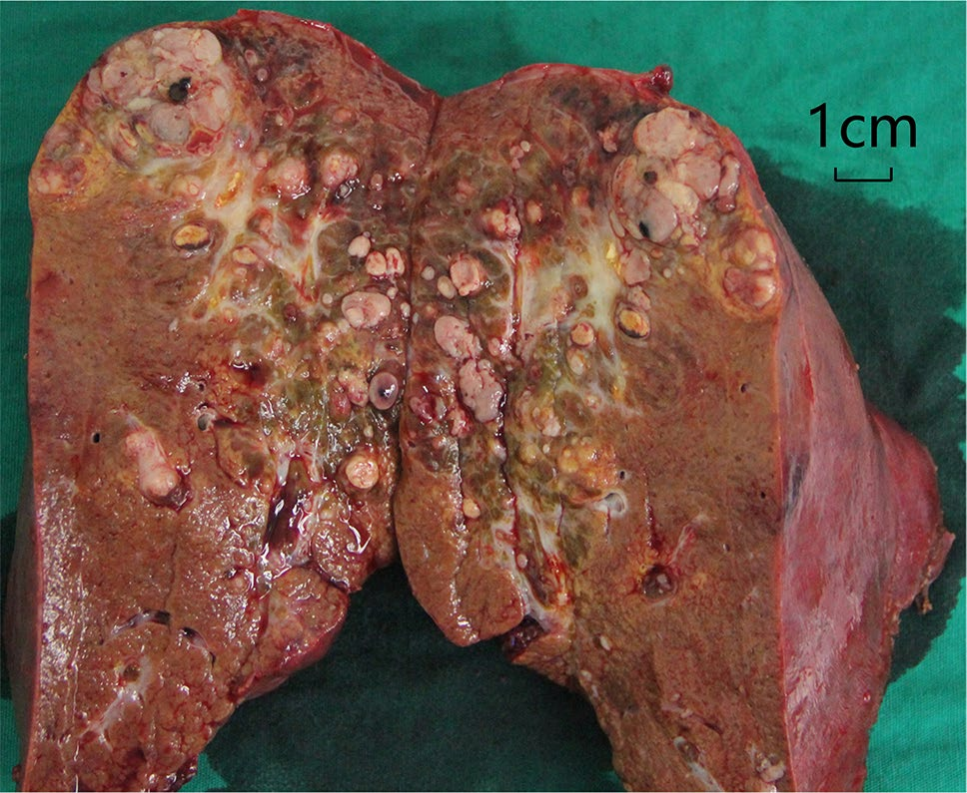
Longitudinal section of the resected right liver lobe. Note the multiple gray-white solid nodules with 0.3–1.2 cm of diameters in size, which displayed well circumscribed or partially encapsulated

**Fig. 3:**
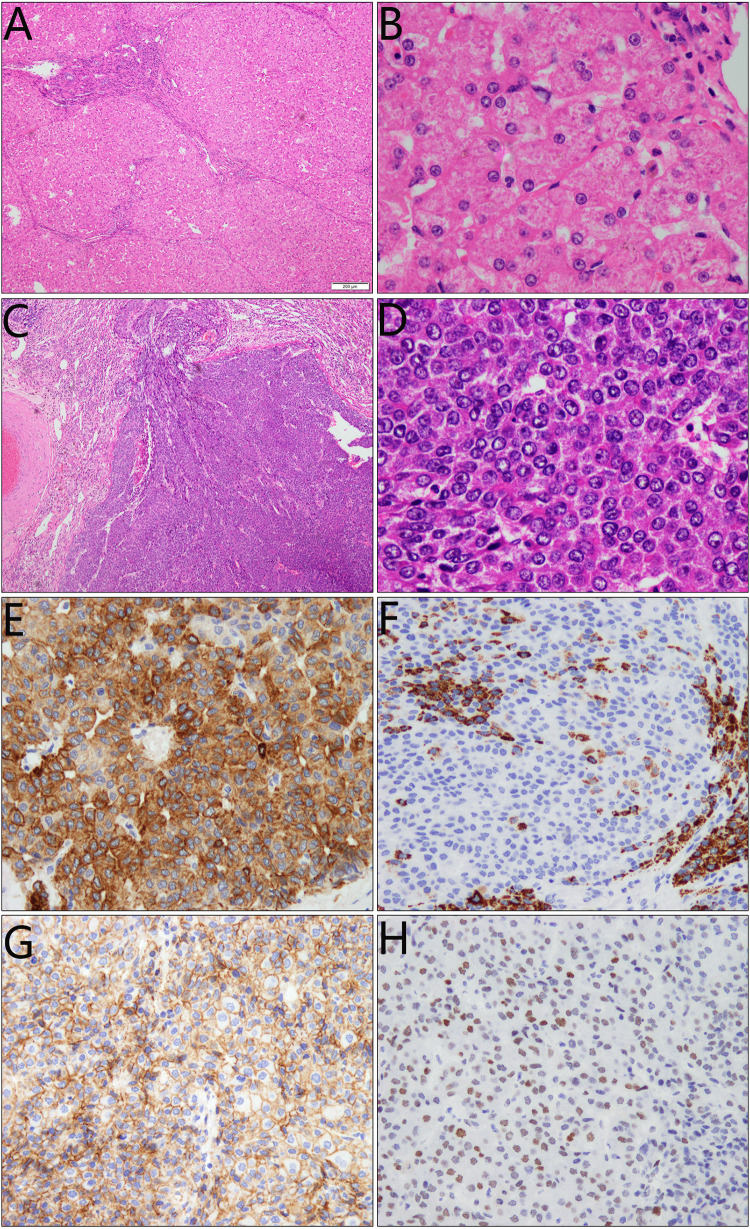
Histopathological findings of the resected liver tissue from the patient with citrin deficiency. Microscopically, multiple nodules were observed in the inflammatory and cirrhotic liver context (**A**, **B**), and the nodules were composed of angulated epithelial-like cells, resembling early-stage differentiation of hepatocytes (**C**). The neoplastic cells arranged in irregular cords and display abundant eosinophilic cytoplasm with indistinct or angulated cellular borders; Nucleus was large or clear with a small nucleolus and less mitoses (**D**). Immunohistochemical staining showed membrane-staining pattern of Cytokeratin 19 (**E**), Hepatocyte (**F**) and Beta-catenin (**G**), along with partially nucleus-staining TP53 (**H**). AFP and Glypican 3 were both positive in a cytoplasmic pattern (not shown)

Her serum AFP level increased rapidly to 134392 ng/ml at the age 4 years and 10 months, when a pre-operative biopsy was conducted on the hepatic mass, and hepatoblastoma was suspected on pathological analysis. The tumor was staged to PRETEXT IV, and the patient underwent neoadjuvant chemotherapy consisting of one course of cisplatin (DDP), two courses of DDP + pirarubicin (THP), and one course of ifosfamide + carboplatin + VP-16(ICE). During the chemotherapy, she experienced recurring anemia, agranulocytosis and thrombocytopenia and was treated symptomatically. As a result, the mass was decreased in size, reaching partial response (PR) on CT-scan under RECIST criteria, and the serum AFP level declined to 25530 ng/ml. This provided an opportunity for surgical resection, and liver transplantation was not immediately considered. Hence, at the age 5 years and 3 months, the patient underwent right hepatic lobectomy and portal venous embolectomy in the Department of Pediatric Surgery, the First Affiliated Hospital, Sun Yat-Sen University. Multiple gray-white solid nodules were observed on a longitudinal section of the resected right hepatic lobe (Fig. 2). Subsequent histopathological analysis of the resected liver and embolus tissues confirmed a fetal epithelial subtype of H, with membrane-staining pattern of Beta-catenin, Cytokeratin 19 and Hepatocyte, along with cytoplasmically positive AFP and Glypican 3, as well as partially nucleus-staining Tumor protein 53(TP53) (Fig. 3). Thereafter, she underwent 5 additional cycles of chemotherapy of ICE regimen, exhibiting a stable postoperative condition for 7 months. Her serum AFP further descended to the level of 5219 ng/ml after surgery and to the lowest 480 ng/ml during the postoperative chemotherapy. However, at the age 5 years and 10 months, her AFP rebounded to 63866 ng/ml, and abdominal ultrasonography revealed “multiple echogenic lesions” in the left liver with the largest one 9 × 7 mm in size, indicating recurred hepatoblastoma. Then her condition deteriorated rapidly into liver failure, and finally led to death at the age 6 years and 1 month.

## Discussion

Although NICCD has been generally regarded as a self-limiting condition, the association of CTLN2 with HCC had been described for years [6–8]. Very recently, Wang et al. reported a pediatric patient with FTTDCD who developed advanced HCC when aged 6 years, according the MRI and CT findings [9]. An additional pediatric patient with FTTDCD reported by He et al. also exhibited MRI feature suggestive of HCC at the age 5 years [10]. These two case reports provided very important evidences supporting the possible linkage of FTTDCD with HCC in pediatric patients. However, due to lacking pathological evidences, such a linkage remains inconclusive. This paper reported a pediatric patient with CD complicated by hepatoblastoma with detailed clinical, molecular and pathological evidences, which was found at the age 4 years and 9 months while led to death at 6 years and 1 month. To the best of our knowledge, this is the first case report addressing the association of CD with hepatoblastoma.

In this paper, the pathological staining findings of Cytokeratin 17, AFP, Hepatocyte and Glypican 3 in the tumor tissue clearly indicated a fetal epithelial subtype of hepatoblastoma [11]. TP53 is unable to bind DNA effectively and loses the tumor-suppressive functions of wild-type P53, thus being associated with an advanced and aggressive tumor phenotype [12–14]. In our patient, the partially positive staining of nuclear TP53 was in consistence with her poor diagnosis. Moreover, beta-catenin usually shows higher intracellular overexpression in most hepatoblastoma cases reported before [15–18]. However, in this hepatoblastoma case, this marker exhibited membrane staining pattern, and this finding suggested that CD might had, although unclear at this time, different tumorigenesis mechanism(s) from traditional hepatoblastoma cases.

Undoubtedly, a solitary case in this paper was not enough to prove the linkage of CD with hepatoblastoma, but either it could not be completely ruled out for CD to serve as one of the underlying mechanisms contributing to hepatoblastoma development. Actually, CD gave rise to citrullinemia, and excessive citrulline had been reported to promote the uptake of thymidine into hepatocytes, which was related to hepatocyte growth and enhancement of DNA synthesis and might lead to liver neoplasms [19]. Besides, impairment of citrin function increased the cytosolic NADH/NAD^+^ ratio of the hepatocyte [10], while oxidative stress, partly attributable to the increased cytosolic NADH [21], could generate reactive oxygen species (ROS) or reactive nitrogen species (RNS) which may contribute to tumorigenesis in CD [22].

Of note, the child in this study displayed remarkable biochemistry changes and marked hepatosplenomegaly when NICCD was diagnosed at her age 10 months, indicating severe liver damage due to an untimely identification of the etiology. Regardless of her initial promising clinical response to dietary therapy, the inflammatory and cirrhotic liver context (Fig. 3A, B) when aged 5 years and 4 months signified a chronic liver injury which might have persisted for years in our patient. The late diagnosis and hence serious liver injury as well as the chronic inflammatory process might also constitute a basis for the formation of her liver hepatoblastoma, necessitating the early etiology diagnosis and close monitoring of the liver indices, morphological and functional, in pediatric CD patients.

In conclusion, this paper, for the first time, described a pediatric CD patient who died of pathologically-confirmed metastatic hepatoblastoma, raising the possible association between the two conditions at pediatric age.

## Data Availability

All datasets generated and analyzed for this study are included in the manuscript. Further inquiries can be directed to the corresponding author.
